# Magnesium Transporters as Crucial Regulators of Bacterial Survival and Pathogenicity

**DOI:** 10.3390/microorganisms14051033

**Published:** 2026-05-01

**Authors:** Seungjun Hur, Youngki Yoo, Jeong Min Chung

**Affiliations:** 1 Department of Biotechnology, The Catholic University of Korea, Bucheon-si 14662, Republic of Korea; hur0948@catholic.ac.kr; 2 Life Sciences Institute, University of Michigan, Ann Arbor, MI 48109, USA; yky@umich.edu

**Keywords:** magnesium transporter, MgtA, MgtB, MgtC, CorA, MgtE, PhoPQ, nutritional immunity, virulence, ESKAPE pathogens

## Abstract

Magnesium is an essential divalent cation required for adenosine triphosphate (ATP)-dependent reactions, nucleic acid metabolism, and ribosomal stability. Bacteria depend on specialized transport systems to maintain intracellular Mg^2+^ homeostasis as it cannot freely cross the phospholipid bilayer. During infection, host nutritional immunity restricts metal availability, and magnesium limitation within the phagosome compromises bacterial metabolism and stability. This review summarizes the major bacterial magnesium transport systems and their roles in survival and pathogenicity, with an emphasis on *Salmonella* and extension to clinically relevant ESKAPE pathogens. We focus on the PhoPQ-regulated MgtA, MgtB, and MgtC system, in which low magnesium, acidic pH, and other host-derived signals activate PhoPQ to induce *mgt* gene expression. MgtA and MgtB act as high-affinity P-type ATPases, whereas MgtC promotes bacterial survival within the intramacrophage environment by inhibiting bacterial F-type ATP synthase through specific interactions with subunit *a*. We also discuss CorA as a conserved channel for basal Mg^2+^ uptake and MgtE as a Mg^2+^-selective channel whose gating responds to intracellular Mg^2+^ and ATP. Finally, we consider the conservation and variation in these systems across pathogenic bacteria and their potential as therapeutic targets for antimicrobial development.

## 1. Introduction

Magnesium is an essential divalent cation and the most abundant free divalent cation in the intracellular environment [1]. It serves as a cofactor in more than 300 enzymatic reactions, including numerous ATP-dependent processes. Intracellular ATP primarily exists as an Mg^2+^–ATP complex, which represents the biologically active form recognized by most ATP-dependent enzymes [2]. DNA and RNA polymerases, as well as nucleases, require Mg^2+^ to catalyze phosphate transfer reactions, where magnesium contributes by stabilizing the active-site structure [3,4].

Magnesium must enter the cytosol through the cell membrane to perform its biological functions. However, the phospholipid bilayer carries a net negative charge, rendering it impermeable to cations [1]. Divalent ions such as magnesium face particular difficulties in crossing the membrane owing to their tightly bound hydration shells [5]. To overcome this barrier, organisms have evolved dedicated magnesium transporters that mediate selective Mg^2+^ uptake into cells.

Magnesium transporters have been identified in various organisms. MgtA/B, CorA, and MgtE are well-characterized Mg^2+^ transporters in bacteria. Each transporter has characteristic structural and regulatory features, but all share the common function of transporting magnesium into cells. The MgtC protein is not directly involved in Mg^2+^ transport, but it contributes to bacterial survival under magnesium-limited conditions. Recent evidence suggests that Mg^2+^ transporters perform additional roles beyond simple ion transport [6,7]. These proteins participate in sensing intracellular Mg^2+^ levels, regulating virulence gene expression, and modulating cellular energy metabolism. In pathogenic bacteria, Mg^2+^ transporters contribute to strategies that overcome host nutritional immunity during infection [8,9].

**Figure 1:**
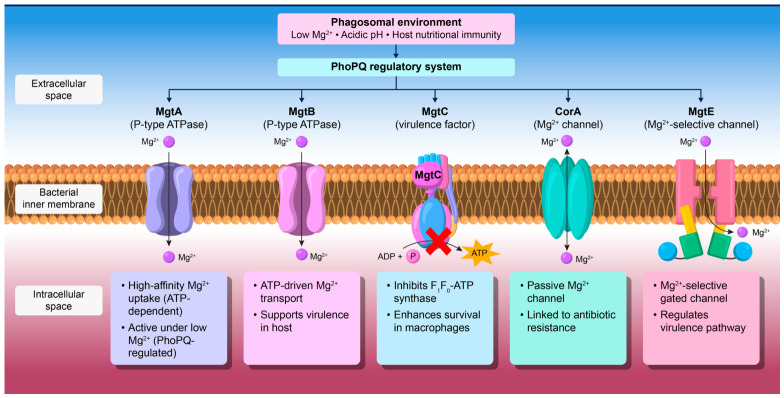
Overview of major bacterial Mg^2+^ transport systems and their roles in survival and pathogenicity. Low Mg^2+^, acidic pH, and host nutritional immunity activate the PhoPQ system, which induces MgtA, MgtB, and MgtC expression. MgtA and MgtB mediate high-affinity Mg^2+^ uptake, whereas MgtC supports bacterial survival in macrophages by modulating ATP synthase activity rather than by directly transporting Mg^2+^. In parallel, CorA functions as a conserved channel for basal Mg^2+^ uptake, and MgtE acts as an Mg^2+^-selective channel regulated by intracellular Mg^2+^ and ATP. This figure summarizes how Mg^2+^ transport and Mg^2+^-responsive systems contribute to bacterial adaptation and virulence.

In this review, we discuss how Mg^2+^ transporters contribute to pathogen survival and virulence, particularly within macrophages. We also discuss recent findings regarding the functions and regulatory mechanisms of MgtA/B/C, CorA, and MgtE (Figure 1). In addition, we examine the conservation and variation in these systems among pathogens and highlight their potential as therapeutic targets for infectious diseases.

## 2. Pathogen Survival in the Phagosome: The MgtA/B/C System

Nutritional immunity is a host defense strategy that restricts the availability of essential metal ions required for pathogen survival, including Fe, Zn, Mn, and Mg^2+^, to suppress microbial function [8]. Magnesium has received less attention than either iron or zinc. However, recent studies have revealed that Mg^2+^ limitation also functions as an important component of nutritional immunity that is closely linked to the regulation of bacterial pathogenicity. Recent studies have provided direct evidence for this mechanism: the host endolysosomal cation channel MCOLN2 has been shown to actively deplete Mg^2+^ from pathogen-containing vacuoles, and ablation of MCOLN2 in human macrophages increased *Salmonella enterica* serovar Typhi intracellular replication by approximately 2.5-fold, confirming that Mg^2+^ deprivation constitutes an active host defense strategy [10]. In addition, cytoplasmic Mg^2+^ starvation has been found to trigger MgtC-dependent restriction of phosphate uptake, thereby limiting ATP precursor availability and coupling Mg^2+^ limitation to global metabolic suppression [11]. Mg^2+^ is essential for various biochemical processes in bacteria, and its limitation leads to ribosomal destabilization, ATP metabolic disruption, and impaired DNA/RNA integrity. Mg^2+^ ions neutralize the negative charges of phosphate groups in the rRNA backbone, and their depletion destabilizes ribosomal subunit assembly [3,4]. At the single-cell level, structural perturbation of the ribosome complex through deletion of ribosomal protein L34 has been shown to increase membrane hyperpolarization events and cell death, and supplementation of exogenous Mg^2+^ was sufficient to suppress hyperpolarization and restore wild-type growth rates, establishing a direct causal relationship between Mg^2+^ availability and ribosome-dependent survival [12]. Consequently, maintaining a low Mg^2+^ concentration within the phagosome alone can markedly reduce bacterial viability.

To counter this host-imposed restriction, pathogens have evolved high-affinity Mg^2^*^+^* transporters, among which the Mgt family has been most extensively characterized, particularly in *Salmonella* and other enteric pathogens, and has been widely studied in the context of intramacrophage survival [5,13]. However, these proteins do not merely function as Mg^2+^ transporters. Instead, they directly influence bacterial survival during infection and regulate virulence gene expression.

### 2.1. Regulation of MgtA/B/C by the PhoPQ Two-Component System

The *mgtA*, *mgtB*, and *mgtC* genes have been most extensively studied in *Salmonella* spp. Therefore, unless otherwise stated, this review primarily refers to the findings derived from *Salmonella*-based studies when discussing the Mgt system. The expression of these genes is tightly regulated by the PhoPQ two-component system, which senses environmental Mg^2+^ levels and serves as a central regulator of bacterial survival strategies [14].

Thus, PhoPQ functions as more than a simple Mg^2+^-sensing system. It acts as a global regulator that coordinates multiple adaptive responses essential for bacterial survival within the macrophages. These responses include resistance to antimicrobial peptides (AMPs), lipopolysaccharide (LPS) modification, and membrane remodeling [15]. For example, PhoPQ regulates the expression of the PmrAB two-component regulatory system and *pagP*, which alter bacterial surface charge to enhance resistance against host cationic antimicrobial peptides (CAMPs) [16,17]. Notably, PhoPQ can sense multiple environmental signals, including low Mg^2+^ concentration and acidic pH [14,17]. Thus, Mg^2+^ limitation and low pH within the phagosomes activate PhoQ. This integrated sensing enables PhoP to precisely adjust gene expression under diverse environmental conditions.

**Figure 2:**
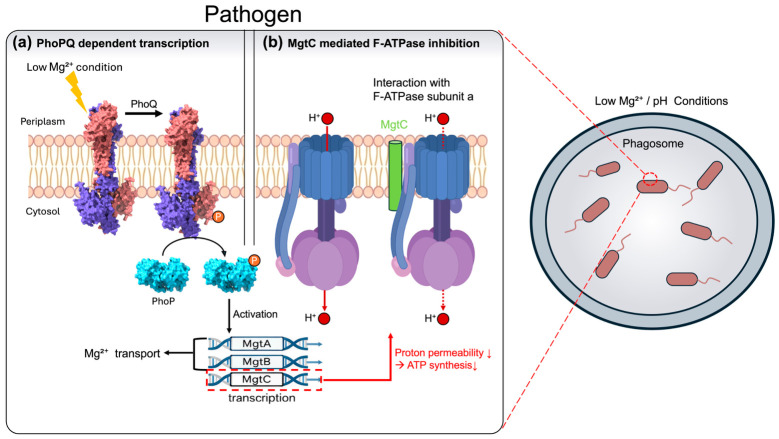
PhoPQ-dependent MgtA, MgtB, and MgtC regulation and MgtC-mediated control of cellular energy metabolism. (**a**) Under low Mg^2+^ conditions and acidic pH characteristic of the phagosome, PhoQ undergoes autophosphorylation and transfers the phosphate group to PhoP. Activated PhoP induces transcription of the *mgtA*, *mgtB*, and *mgtC* genes. MgtA and MgtB function as Mg^2+^ transporters and contribute to the maintenance of intracellular Mg^2+^ homeostasis. (**b**) MgtC interacts specifically with subunit *a* of the F_1_F_o_ ATP synthase and reduces ATP synthesis, thereby modulating cellular energy metabolism. This combined regulation supports bacterial survival under low-Mg^2+^ and low-pH conditions within the phagosome.

PhoQ is a histidine kinase located in the bacterial inner membrane. Under low external Mg^2+^ conditions, PhoQ undergoes autophosphorylation and subsequently transfers the phosphate group to the cytoplasmic response regulator PhoP, thereby activating it [18] (Figure 2). Activated PhoP promotes the transcription of numerous genes, including *mgtA*, *mgtB*, and *mgtC*. Thus, the PhoPQ system governs not only Mg^2+^ transporter expression but also multiple survival mechanisms, such as antimicrobial peptide resistance, membrane remodeling, and LPS modification, all of which are essential for successful infection [15] (Figure 2).

### 2.2. MgtA and MgtB: P-Type ATPases with Conserved Architecture

**Figure 3:**
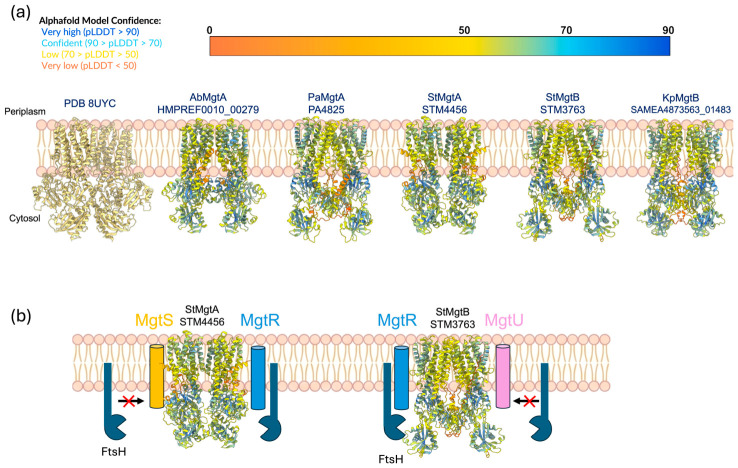
Structural comparison of MgtA and MgtB and their regulation by small membrane peptides. (**a**) Experimentally determined structure of MgtA (PDB 8UYC) and AlphaFold-predicted models of MgtA and MgtB from different bacterial species in a membrane context. Models are colored according to AlphaFold confidence scores (pLDDT). Despite sharing approximately 50% sequence similarity, MgtA and MgtB display highly similar overall architectures, including conserved transmembrane and cytosolic domains, supporting their classification as closely related P-type ATPases. (**b**) Schematic representation of the differential regulation of MgtA and MgtB by small membrane peptides and the FtsH protease. MgtA is stabilized by interaction with MgtS, whereas MgtB is stabilized by interaction with MgtU, protecting each transporter from FtsH-mediated degradation. In contrast, the regulatory peptide MgtR promotes FtsH-dependent degradation of both MgtA and MgtB. This peptide-mediated control provides a mechanism to tune the cellular abundance of MgtA and MgtB in response to Mg^2+^ availability and other physiological conditions.

MgtA and MgtB are P-type ATPases that import Mg^2+^ into the cells with a high affinity through ATP hydrolysis. These proteins share approximately 50% sequence similarity [5]. The structural predictions generated by AlphaFold suggest that they possess similar architectures overall. Despite their structural resemblance, they exhibit distinct functional properties, particularly in their interactions with small membrane proteins. MgtA associates with MgtS, whereas MgtB interacts with MgtU. Specifically, MgtS is a small inner membrane peptide (31 amino acids) that stabilizes MgtA by directly interacting with its transmembrane domain, thereby preventing FtsH-mediated proteolytic degradation [19]. Similarly, MgtU plays an analogous protective role for MgtB. In the absence of MgtS or MgtU, the respective transporter is rapidly degraded, leading to reduced intracellular Mg^2+^ uptake and compromised bacterial survival within macrophages [19] (Figure 3). In contrast, both MgtA and MgtB interact with a regulatory peptide, MgtR, which enhances their susceptibility to FtsH-mediated degradation [20]. This regulatory mechanism enables bacteria to modulate transporter abundance in response to changes in magnesium levels. Recent studies suggest that the activities of these transporters can also vary depending on specific lipid compositions of the membrane, as MgtA has been shown to be activated by cardiolipin and is sensitive to free Mg^2+^ concentrations in vitro [21,22]. Together, these findings indicate that MgtA and MgtB are not merely simple ion pumps; instead, they are highly regulated transporters sensitive to various physiological factors, including membrane composition, protein complex formation, and cellular energy status.

In particular, MgtB is known to function as a direct regulator of virulence in several pathogens. In *Yersinia* species, MgtB enhances bacterial survival during the early stages of infection, and mgtB deletion markedly reduces virulence [23]. Moreover, studies in *Salmonella* have demonstrated that MgtB plays a critical role in maintaining virulence within macrophages expressing functional Slc11a1^+/+^ (also known as NRAMP1), a proton-coupled divalent cation transporter localized to the phagosomal membrane. Slc11a1 mediates pH-dependent efflux of Fe^2+^, Mn^2+^, and other divalent cations from the acidified phagosomal lumen into the cytoplasm, thereby imposing severe nutritional stress on intracellular pathogens [24,25]. Under these conditions, the absence of MgtB drastically decreases bacterial survival, underscoring its essential role in maintaining Mg^2+^ homeostasis during extreme nutritional stress.

Recent structural studies have revealed that MgtA, which shares high similarity with MgtB, was long thought to function as a monomer but instead forms a stable homodimer at 2.9 Å resolution, the first dimeric structure reported for any P-type ATPase [26]. This observation suggests that the regulatory mechanisms governing MgtA and MgtB function are more complex than previously appreciated, emphasizing the need for further structural investigation.

### 2.3. MgtC: A Virulence Factor That Does Not Transport Mg^2+^

MgtC does not directly transport Mg^2+^ but functions as an atypical virulence protein critical for bacterial survival under Mg^2+^-limiting conditions within macrophages [6,27]. Similar to MgtA and MgtB, MgtC is degraded by the FtsH protease upon its interaction with the regulatory peptide MgtR [28]. Recent findings have revealed that MgtC directly interacts with subunit a of the F_1_F_o_ ATP synthase to inhibit bacterial ATP synthesis [7]. This inhibition represents a survival strategy that enables bacteria to minimize ATP consumption and prioritize survival under energy-limited conditions such as those inside macrophages.

MgtC sustains PhoPQ activation through two distinct feedback mechanisms. First, reduced ATP levels further suppress the activity of ATP-dependent proteases (e.g., ClpS-ClpAP), thereby stabilizing regulatory proteins, such as PhoP against proteolysis [29]. Stabilization of PhoP subsequently leads to sustained *mgtA/B/C* expression, allowing MgtC to maintain PhoPQ activation through an indirect feedback loop [30].

Second, and independently, MgtC directly interacts with PhoP, promoting PhoP stability and creating a direct positive feedback loop to sustain transcription of *mgtC* itself, as well as *mgtA* and *mgtB* [13]. These regulatory mechanisms demonstrate that MgtC functions not as a single virulence factor but as a higher-order regulator coordinating bacterial survival under host-imposed stress [7].

MgtC has been predominantly identified in pathogenic bacteria and is rarely found or expressed in non-pathogenic strains [6,13,27]. This pattern suggests that MgtC has evolved to specialize in infection environments, highlighting its potential as a future therapeutic target against bacterial pathogenesis.

### 2.4. Mgt Homolog Distribution in Clinically Relevant Pathogens Beyond Salmonella

To date, MgtA/B/C has mainly been studied in *Salmonella* spp. However, genomic analyses (Table 1) have indicated the presence of Mgt homologs in various other pathogenic bacteria. Notably, the distribution of individual Mgt components varied across the species. Although *Pseudomonas aeruginosa* (*P. aeruginosa*) and *Acinetobacter baumannii* (*A. baumannii*) harbor MgtA and MgtC homologs, they lack a canonical MgtB ortholog, contrasting with the intact *mgtCB* operon in *Salmonella*. This raises intriguing evolutionary questions regarding the selective pressures that shaped the Mgt system in different pathogenic contexts. These pathogens belong to the clinically significant and antibiotic-resistant ESKAPE pathogen group. However, the structural features, regulatory mechanisms, interacting partners, and membrane lipid composition of the Mgt system in these non-*Salmonella* pathogens remain unknown. Further research into these areas will provide valuable insights into the conservation and pathogen-specific variations in the Mgt system, and potentially uncover novel targets for antimicrobial therapies. To complement the strain-level summary in Table 1, comparative phylogenetic analyses of MgtA/MgtB and MgtC homologs across these pathogens are provided as Supplementary Figure S1, with the underlying multiple sequence alignments included as Supplementary Data S1 and S2. A broader-scale phylogenetic analysis of MgtA homologs from 2450 bacterial strains, together with the corresponding sequence metadata, is provided as Supplementary Data S3-1 and S3-2.

## 3. CorA: A Conserved Mg^2+^ Channel with Emerging Roles in Pathogenesis

CorA is a highly conserved Mg^2+^ transporter, found in nearly all prokaryotic species [35]. Homologous proteins, such as Mrs2, are also present in the inner membrane of mitochondria in some eukaryotic cells. This high degree of conservation suggests that CorA is a fundamental transporter required for basic cellular functions. The physiological roles of CorA have been verified in a wide range of bacterial species, including *Escherichia coli* (*E. coli*), *Salmonella*, *Mycobacterium*, *Bacillus subtilis*, and *Pseudomonas aeruginosa* [36,37]. CorA functions as a transmembrane channel that facilitates the passive influx of Mg^2+^ into the cell, driven by an electrochemical gradient. This process does not require an energy input and occurs only when extracellular Mg^2+^ concentrations exceed intracellular levels. As such, CorA serves as the primary transporter for satisfying basal Mg^2+^ requirements under normal growth conditions [35].

CorA typically forms a pentameric complex in the membrane. Each subunit contains one transmembrane domain and one cytoplasmic regulatory domain [38]. Channel gating is controlled by Mg^2+^ binding to the cytoplasmic domain via a negative feedback mechanism. When the intracellular Mg^2+^ concentration increases, the channel closes, and when Mg^2+^ levels decrease, the channel opens [39]. The GxN (or GMN) motif plays a central role in both Mg^2+^ binding and gate regulation [40]. This motif allows the selective transport of divalent cations, including Mg^2+^. Binding of Mg^2+^ causes the channel to constrict, blocking ion flow, whereas Mg^2+^ release expands the channel, permitting influx. This structural regulation allows CorA to maintain intracellular Mg^2+^ homeostasis without energy expenditure, making it particularly advantageous under low-energy conditions [35,38].

CorA has traditionally been viewed as an essential transporter for core bacterial physiology. However, emerging evidence shows that CorA also participates in infection and pathogenesis. In *Salmonella enterica* serovar Typhimurium (*S.* Typhimurium), a single deletion of *corA* produces a relatively mild phenotype, likely because MgtA and the PhoPQ system provide compensatory Mg^2+^ uptake. However, the combined deletion of *corA* and *phoP* produces a synthetic genetic effect, resulting in marked growth reduction, diminished motility, and competitive disadvantage [41]. This indicates that CorA- and PhoPQ-regulated transporters function as complementary Mg^2+^ acquisition pathways. Additionally, CorA has been reported to mediate the nonselective transport of other divalent metal ions, such as Ca^2+^, Co^2+^, and Ni^2+^, in species including *Pectobacterium versatile*, *Salmonella enterica*, and *E. coli* [36,42]. These properties suggest that CorA contributes to maintaining ionic balance in complex infection environments. Such versatility implies that CorA functions not only in Mg^2+^ transport but also in adaptation to environmental stressors such as membrane stability, osmotic pressure, and pH fluctuations.

Notably, a recent study has revealed an association between CorA and antibiotic resistance. A recent study [43] revealed that *corA* deletion in *Mycobacterium smegmatis* increased its sensitivity to structurally diverse antibiotics. Resistance to fluoroquinolones and aminoglycoside antibiotics was also reduced. This phenotype was also observed in *E. coli* recombinantly expressing *M. smegmatis* CorA. These results suggest that CorA acts as a structural platform to maintain the function of antibiotic efflux systems. Although *M. smegmatis* is a non-pathogenic model organism, these findings have significant implications for pathogenic mycobacteria such as *M. tuberculosis*, where CorA homologs may similarly influence intrinsic antibiotic resistance. Future studies using pathogenic mycobacterial strains will be critical to validate this hypothesis.

CorA is highly conserved not only in pathogenic bacteria but also in non-pathogenic and commensal strains. This conservation indicates that CorA is a fundamental component of bacterial survival and is not restricted to virulent species [35]. However, in pathogenic contexts, CorA may interact with virulence regulatory systems to support adaptation to changing environmental conditions. Further studies are required to characterize CorA’s structural stability, ion selectivity, contribution to antibiotic sensitivity, and potential interplay with other transporters during infection. Defining CorA as a virulence accessory factor and exploring its role in antibiotic resistance mechanisms may open new opportunities for antimicrobial development.

## 4. MgtE: A Mg^2+^ Channel That Regulates Pathogenicity

MgtE is a Mg^2+^ channel widely conserved in both prokaryotes and eukaryotes [44]. The MgtE channel of bacteria is highly selective for Mg^2+^ and is involved in maintaining intracellular Mg^2+^ concentrations [45]. Structurally, MgtE is not an ATPase but rather an Mg^2+^-selective channel whose gating is regulated through structural changes induced by ATP binding [46]. When the intracellular Mg^2+^ and ATP concentrations are high, ATP binds to MgtE and stabilizes its closed conformation. This closed state prevents Mg^2+^ influx by obstructing the ion conduction pathway. Conversely, when intracellular Mg^2+^ and ATP concentrations are low, ATP dissociates from MgtE, resulting in the structural relaxation of the cytosolic domain and opening of the transport channel. These structural transitions occur without direct energy expenditure and allow MgtE to actively respond to fluctuations in cellular Mg^2+^ and ATP concentrations [46]. Crystallographic analysis of the MgtE cytoplasmic domain in complex with ATP has revealed that ATP coordinates between the N-terminal and CBS domains, inducing a conformational closure that directly occludes the ion conduction pathway [46]. Complementary cryo-EM analysis of full-length MgtE in the Mg^2+^-free state at near-atomic resolution further elucidated the open-to-closed gating transition, with specific rearrangements in TM2 and TM5 facilitating channel closure upon ATP binding [47].

MgtE of *P. aeruginosa* has been shown to function as more than a simple Mg^2+^ transporter and directly participates in infection persistence and pathogenicity [48,49]. The mutant strain lacking the *mgtE* gene showed altered phenotypes associated with cytotoxicity, including changes in exoenzyme expression and the regulation of biofilm-associated genes. It has been suggested that cytotoxicity regulation via Type III secretion system (T3SS) suppression could represent a critical step in chronic *P. aeruginosa* infections [48,49]. A previous study demonstrated gradual T3SS suppression following airway infection [50], and this T3SS regulation appears to be mediated by MgtE.

Co-culture experiments with *P. aeruginosa* and human-derived cystic fibrosis bronchial epithelial (CFBE) cells further demonstrated that MgtE deficiency significantly influenced T3SS expression, exoenzyme production, and biofilm-associated gene regulation, thereby altering bacterial cytotoxicity [51]. This finding suggests that MgtE is a regulatory factor closely integrated into the bacterial virulence network, extending its role beyond Mg^2+^ transport to include the direct modulation of pathogenic mechanisms essential for establishing chronic infections.

## 5. Mg^2+^ Transporters as Potential Therapeutic Targets

Mg^2+^ transport-related systems deserve attention as potential drug targets because their functions extend beyond Mg^2+^ uptake to directly support bacterial survival and virulence in host environments. Several features support their potential as therapeutic targets.

First, MgtC, for example, enhances bacterial survival within macrophages by altering cellular energy metabolism and helping pathogens withstand Mg^2+^-limited intracellular stress. As a result, it contributes to persistence in immune cells, which is a key feature of pathogenicity in several bacteria. MgtC is predominantly found in pathogenic bacteria and has no characterized mammalian counterpart, and the bacterial F_1_F_o_ ATP synthase subunit *a* targeted by MgtC differs substantially from the mitochondrial ATP synthase, reducing the likelihood of off-target effects. Cryo-EM studies have revealed that the bacterial F_o_ region consists of only three core subunits (ab_2_c_9–15_), in contrast to the more elaborate organization of the mammalian mitochondrial enzyme, which includes numerous supernumerary subunits absent from bacteria [52]. Moreover, a recent high-resolution (2.0–2.4 Å) structure of the *P. aeruginosa* ATP synthase identified species-specific features in the F_o_ region, including a unique ε-subunit inhibitory binding site and a coordinated metal ion (identified as zinc by mass spectrometry) capping the cytoplasmic proton channel, neither of which is found in mitochondrial counterparts [53]. These structural divergences support the feasibility of selective inhibitor design targeting the MgtC–ATP synthase interaction interface.

Second, the availability of high-resolution cryo-EM structures, including the recent MgtA dimer structure at 2.9 Å resolution [26] and the well-characterized CorA pentameric channel [38,39], provides a foundation for structure-based drug design (SBDD) approaches. Computational screening of compound libraries against MgtA’s ATP-binding site or the MgtC–F_1_F_o_ interaction interface could identify lead compounds for further development.

Third, the association between CorA and intrinsic antibiotic resistance in mycobacteria [43] suggests that targeting CorA function could serve as an adjuvant strategy to potentiate the activity of existing antibiotics. MgtE also appears to have consequences beyond Mg^2+^ homeostasis, as it has been linked to changes in cytotoxicity and T3SS regulation. Because these proteins influence infection-relevant outcomes, their inhibition could lead not only to impaired adaptation to host-imposed stress but also to the attenuation of bacterial virulence and persistence.

Although no specific inhibitors of bacterial Mg^2+^ transporters have yet advanced to clinical development, the convergence of structural data, infection-specific expression profiles, and validated virulence phenotypes positions this transporter family as a promising frontier for next-generation antimicrobial discovery.

## 6. Conclusions and Future Perspectives

Mg^2+^ is an essential metal ion required for the survival of all living organisms. Mg^2+^ is involved in almost all physiological processes, including cellular metabolism, gene expression, protein translation, and energy production. Because Mg^2+^ cannot pass through the cell membrane spontaneously, organisms have evolved specialized transporters to facilitate its selective uptake. Importantly, these transporters are now increasingly recognized not only for their role in ion transport but also for their direct involvement in bacterial survival and virulence under infection conditions.

This review focuses on the major Mg^2+^ transporters, namely MgtA, MgtB, MgtC, CorA, and MgtE, and examines their biochemical properties, structures, regulatory mechanisms, and functional diversity in pathogenic bacteria. Their roles are crucial for bacterial adaptation to the Mg^2+^-limited phagosomal environment within macrophages. MgtA and MgtB are high-affinity P-type ATPases expressed under the control of the PhoPQ system. They import Mg^2+^ into cells under low-Mg^2+^ conditions, supporting bacterial survival. Their protein stability is regulated through interactions with small regulatory peptides, and they are also responsive to specific membrane lipid compositions. MgtB has been shown to directly influence virulence in *Yersinia* and *Salmonella*. MgtB in *Salmonella* functions as an essential factor for survival in Slc11a1^+/+^ macrophages.

MgtC does not directly transport Mg^2+^ but acts as a strategic factor by suppressing ATP synthesis through interaction with subunit *a* of the F_1_F_o_ ATP synthase, stabilizing PhoP, and maintaining the PhoPQ system through dual positive feedback loops. This suggests that MgtC functions not merely as a virulence factor but as a higher-order regulator orchestrating bacterial survival strategies. CorA is a conserved pentameric channel for maintaining intracellular Mg^2+^ homeostasis. It is highly conserved in structure and operates through gating controlled by the GxN motif. Recent findings indicate that CorA in *Mycobacterium smegmatis* can influence antibiotic susceptibility, suggesting that it may also contribute to infection adaptation and resistance regulation. Validation in pathogenic mycobacterial species remains an important next step.

As discussed in Section 4, MgtE regulates its gating through ATP binding rather than hydrolysis. In *P. aeruginosa*, MgtE has been linked to T3SS regulation and infection-mode switching, positioning it as a multifunctional virulence-associated factor beyond its canonical role in Mg^2+^ transport. The structural, functional, and regulatory characteristics of the major bacterial Mg^2+^ transporters discussed in this review are summarized in Table 2.

These findings demonstrate that Mg^2+^ transporters play a wide range of roles that extend beyond basic survival. They contribute to virulence expression, infection persistence, and regulation of antibiotic resistance, highlighting their biological significance and potential as therapeutic targets. If transporters that are specifically expressed or activated in infection environments can be precisely inhibited, it may be possible to suppress pathogen survival while minimizing disruption to the host microbiota. This could provide a foundation for next-generation precision antimicrobial strategies.

However, the mechanisms by which these Mg^2+^ transporters are connected to virulence regulation remain incompletely understood. Further investigation is needed to clarify their regulatory elements, structure-based activation mechanisms, and pathogen-specific expression patterns. Multifunctional regulators like MgtC and MgtE, which extend beyond traditional transporter definitions, may become key research topics in future studies on infectious diseases. Ongoing research should include comparative analyses of transporter function across different pathogens, mapping of regulatory networks, and structure-based inhibitor design. These efforts will not only contribute to the control of infectious diseases but also provide important insights into the evolutionary dynamics of bacterial virulence.

## Figures and Tables

**Table 1:** Distribution of MgtA, MgtB, and MgtC homologs in pathogenic bacteria. The table summarizes representative MgtA, MgtB, and MgtC homologs identified in pathogenic bacteria, including members of the ESKAPE pathogen group such as *Pseudomonas aeruginosa*, *Acinetobacter baumannii*, *Enterococcus faecium*, and *Klebsiella pneumoniae*. For each strain, the annotated protein name, UniProt accession number, and corresponding gene (ORF) identifier are listed [19,26,31,32,33,34].

Strain	Protein	UniProt ID	Gene (ORF)
*P. aeruginosa (PAO1)*	MgtA	Q9HUY5	PA4825
	MgtC	Q9I0S6	PA2558
	MgtC	Q9HVF6	PA4635
	MgtC	Q9I1W7	PA2148
*A. baumannii (ATCC 19606)*	MgtA	D0C699/A0ABX6CGY9	HMPREF0010_00279
	MgtC	D0C6A0/A0ABX6CGZ0	HMPREF0010_00280
*E. faecium (BAA-472)*	MgtA	Q3Y0B4	HMPREF0351_10642
	MgtC	Q3Y0E8	HMPREF0351_12381
*S. typhimurium (LT2)*	MgtA	P36640	STM4456
	MgtB	P22036	STM3763
	MgtC	P0CI70	STM3764
*K. pneumoniae (NCTC 13443)*	MgtA	A0A086ICS8	NCTC13443_01257
*K. pneumoniae (5012STDY7626362)*	MgtB	A0A486UQ81	SAMEA4873563_01483
*K. pneumoniae (IS43)*	MgtC	W1DJ03	PRK15385

**Table 2:** Comparison of major bacterial Mg^2+^ transporters.

Feature	MgtA	MgtB	MgtC	CorA	MgtE
Type	P-type ATPase	P-type ATPase	Virulence protein	Pentameric channel	Mg^2+^-selective channel
Energy	ATP hydrolysis	ATP hydrolysis	N/A (inhibits F_1_F_o_)	Passive	Passive (ATP-gated)
Signal	Low Mg^2+^, pH (PhoPQ)	Low Mg^2+^, pH (PhoPQ)	Low Mg^2+^, pH (PhoPQ)	Intracellular Mg^2+^	Intracellular Mg^2+^/ATP
Regulation	PhoPQ; MgtS/MgtR; FtsH	PhoPQ; MgtU/MgtR; FtsH	PhoPQ; MgtR/FtsH; PhoP loops	GxN motif gating	ATP-binding gating
Virulence role	Mg^2+^ uptake in phagosome	Virulence (Yersinia, Salmonella)	F_1_F_o_ inhibition; PhoP stab.	Antibiotic resistance	T3SS regulation
Key pathogens	*Salmonella*, *E. coli*, *P. aeruginosa*, *A. baumannii*	*Salmonella*, *Yersinia*, *K. pneumoniae*	*Salmonella*, *P. aeruginosa*, *A. baumannii*	Nearly all prokaryotes	*P. aeruginosa*
Structure	Cryo-EM dimer/monomer (8UYC) [26]	AlphaFold models	No high-res structure, AlphaFold models	X-ray crystal [38]/Cryo-EM [54] pentamer	X-ray crystal [45]/Cryo-EM structure binding with Fab [47]

## Data Availability

All data supporting this review are contained within the article and the Supplementary Materials. No new primary datasets were generated.

## Supplementary Materials

The following supporting information can be downloaded at: https://www.mdpi.com/article/10.3390/microorganisms14051033/s1, Supplementary Figure S1: Phylogenetic relationships of MgtA/MgtB and MgtC homologs in pathogenic bacteria; Supplementary Data S1: Multiple sequence alignment of MgtA and MgtB homologs from pathogenic bacteria; Supplementary Data S2: Multiple sequence alignment of MgtC homologs from pathogenic bacteria; Supplementary Data S3-1: Broad-scale phylogenetic tree of MgtA homologs from 2450 bacterial strains; Supplementary Data S3-2: Metadata for MgtA homolog sequences used in the broad-scale phylogenetic analysis.

## Abbreviations

AMP	Antimicrobial peptide
ATP	Adenosine triphosphate
CAMPs	Cationic antimicrobial peptides
CFBE	Cystic fibrosis bronchial epithelial
ESKAPE	*Enterococcus faecium*, *Staphylococcus aureus*, *Klebsiella pneumoniae*, *Acinetobacter baumannii*, *Pseudomonas aeruginosa*, and *Enterobacter species*
LPS	Lipopolysaccharide
PDB	Protein Data Bank
pLDDT	Predicted local distance difference test
SBDD	Structure-based drug design
T3SS	Type III secretion system
