# Supplementary material for: Magnesium Transporters as Crucial Regulators of Bacterial Survival and Pathogenicity
Source: Microorganisms. 2026 May 1;14(5):1033. doi: 10.3390/microorganisms14051033 (PMC13209247; doi:10.3390/microorganisms14051033)
Supplement: Supplementary file 1 [file microorganisms-14-01033-s001.zip › microorganisms-4251839-supplementary/Supplementary_materials/Supplementary_Data_Legends.pdf]

**Legends for Supplementary Materials**

**Supplementary Data S1. Multiple sequence alignment of MgtA and MgtB homologs from pathogenic bacteria.** Jalview alignment view (PNG image) of MgtA and MgtB homologs used as input for the phylogenetic analysis shown in Supplementary Figure 1a, colored by percentage identity. The dataset includes the following strains and UniProt accessions: *Acinetobacter baumannii* MDR-ZJ06 (A0A654L116), *A. baumannii* SDF (B0VRM8), *A. baumannii* ATCC 19606 (D0C699), *Escherichia coli* K-12 (P0ABB8), *E. coli* O44:H18 EAEC (D3GW42), *Yersinia pestis* (A0AAX2HZE9), *Pseudomonas aeruginosa* PAO1 (Q9HUY5), *P. aeruginosa* UCBPP-PA14 (A0A0H2ZH03), *P. aeruginosa* PA7 (A6VCT0), *Salmonella enterica* serovar Typhimurium LT2 (MgtA: P36640; MgtB: P22036), *S.* Typhimurium 14028s (D0ZTB2), *S. enterica* (A0A0W3KK13), *Klebsiella pneumoniae* (A0A086ICS8 / A0A486UQ81), and *Enterococcus faecium* ATCC BAA-472 (Q3Y0B4). *E. coli* K-12 was included as a non-pathogenic reference for comparison with pathogenic *E. coli*. Although sequence divergence is evident among species, multiple conserved regions are apparent across the alignment, supporting the shared assignment of MgtA and MgtB to the $Mg^{2+}$-transporting P-type ATPase family.

**Supplementary Data S2. Multiple sequence alignment of MgtC homologs from pathogenic bacteria.** Jalview alignment view (PNG image) of MgtC homologs used as input for the phylogenetic analysis shown in Supplementary Figure 1b, colored by percentage identity. The dataset includes the following strains and UniProt accessions: *S.* Typhimurium LT2 (P0CI70), *S.* Typhimurium 14028s (D0ZLQ7), *S. enterica* (A0A0M0QP52), *P. aeruginosa* UCBPP-PA14 (paralogs A0A0H2ZHS2 / A0A0H2Z9J0 / A0A0H2ZAK6), *P. aeruginosa* PAO1 (paralogs Q9HVF6 / Q9I0S6 / Q9I1W7), *P. aeruginosa* PA7 (paralogs A6V4Q2 / A6V633 / A6VC28), *K. pneumoniae* IS43 (W1DJ03), *A. baumannii* ATCC 19606 (D0C6A0), and *A. baumannii* ATCC 17978 (A0A077GEF9). The alignment shows residue-level conservation and divergence within the MgtC homolog set across these pathogens.

**Supplementary Data S3-1. Broad-scale phylogenetic tree of MgtA homologs from 2,450 bacterial strains.** Distance-based phylogenetic tree (PDF) inferred from a Clustal Omega multiple sequence alignment of MgtA homologs collected from 2,450 bacterial strains, visualized as a circular cladogram. Strains were randomly selected from UniProt entries annotated as carrying MgtA. The corresponding sequence metadata are provided in Supplementary Data 3-2.

**Supplementary Data S3-2. Metadata for MgtA homolog sequences used in the broad-scale phylogenetic analysis.** Excel file containing the sequence information for the MgtA homologs analyzed in Supplementary Data 3-1. Sheet 1 lists the FASTA-formatted MgtA sequences used as the input for Clustal Omega multiple sequence alignment. Sheet 2 provides the corresponding curated metadata table, including UniProt accession, annotated protein name, and source organism for each of the 2,450 entries.
