# Supplementary material for: Magnesium Transporters as Crucial Regulators of Bacterial Survival and Pathogenicity
Source: Microorganisms. 2026 May 1;14(5):1033. doi: 10.3390/microorganisms14051033 (PMC13209247; doi:10.3390/microorganisms14051033)
Supplement: Supplementary file 1 [file microorganisms-14-01033-s001.zip › microorganisms-4251839-supplementary/Supplementary_materials/Supplementary_Figure_S1.pdf]

Supplementary Figure

(a)

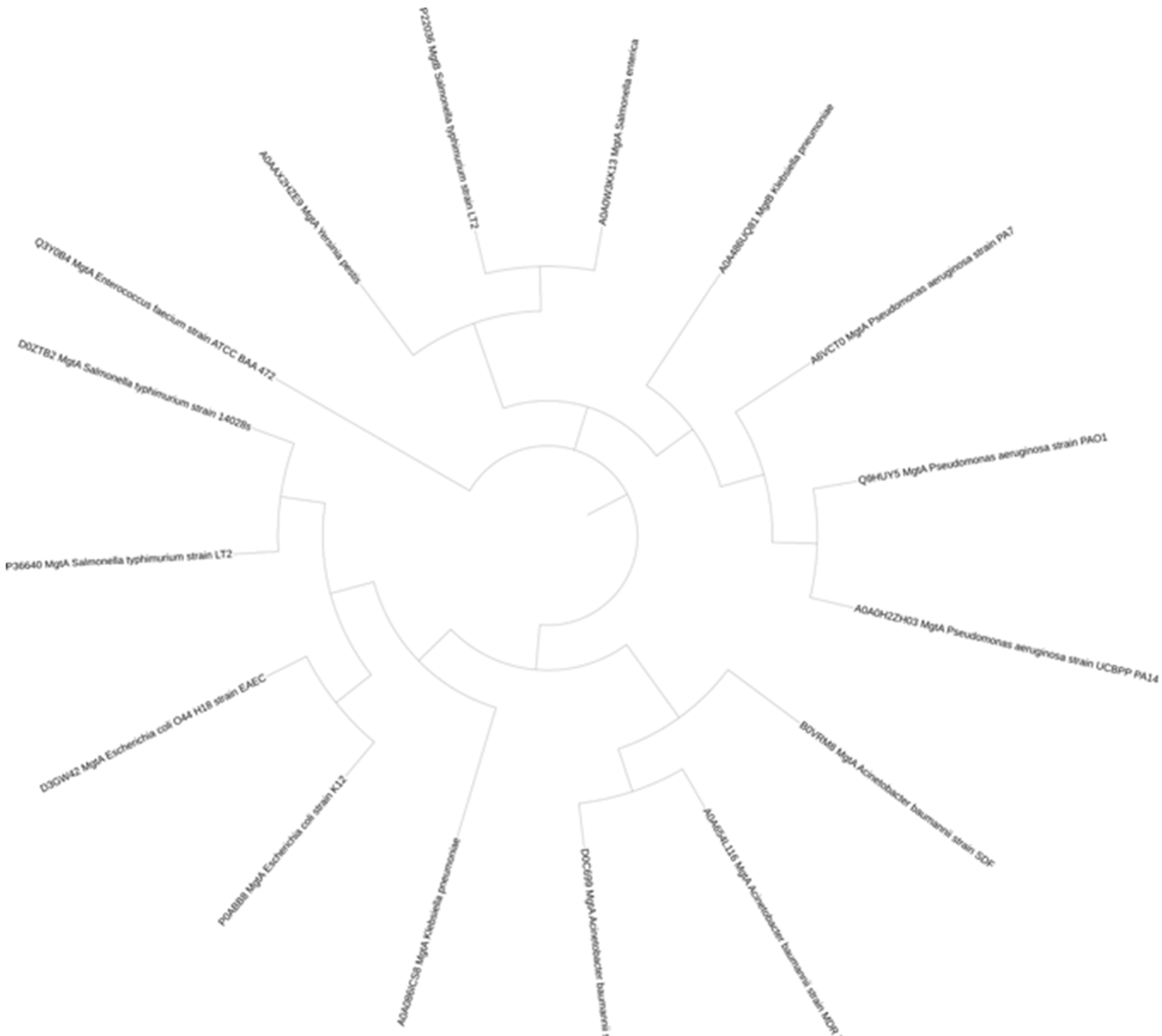

(b)

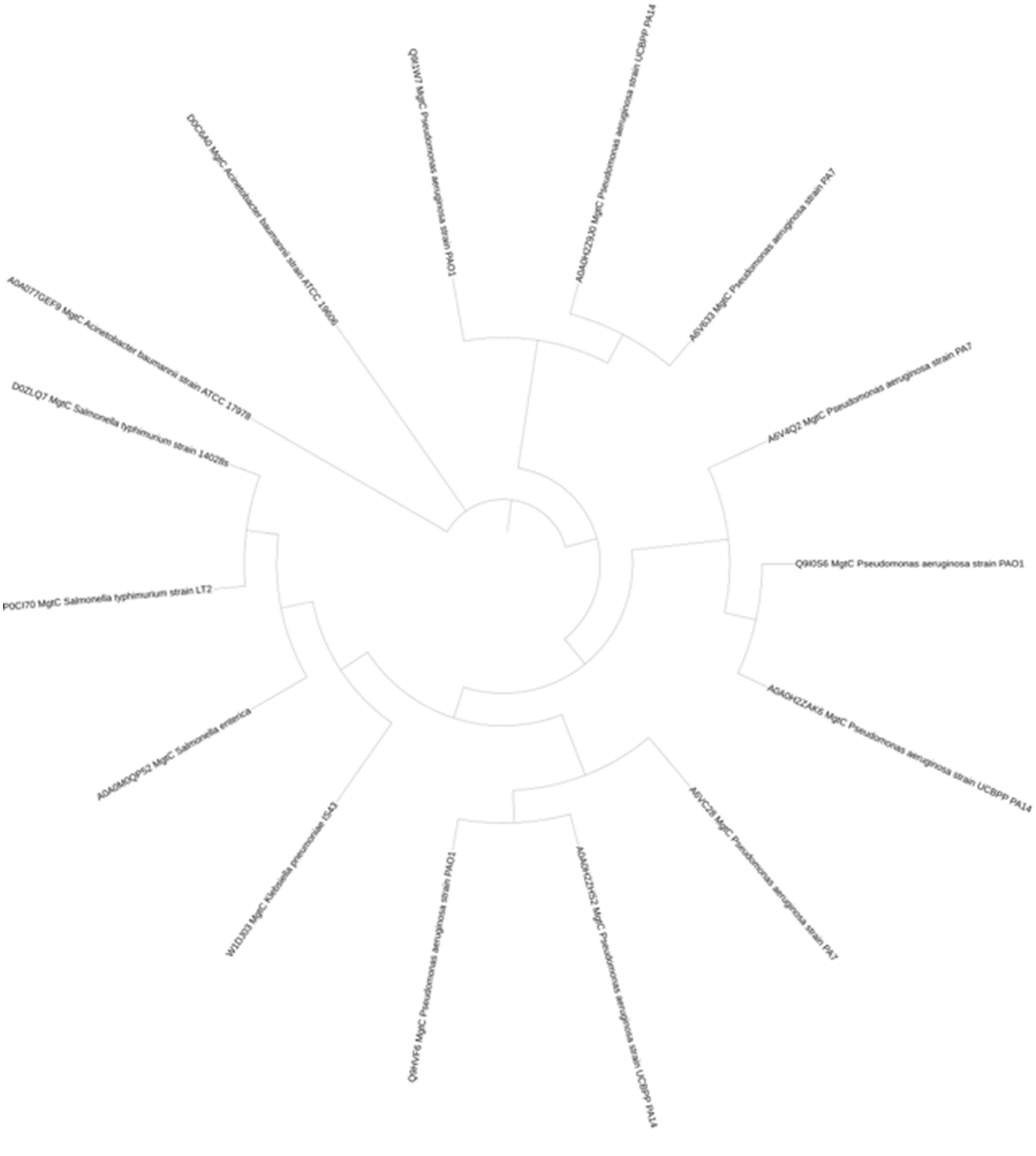

Supplementary Figure S1. Phylogenetic relationships of MgtA/MgtB and MgtC homologs in pathogenic bacteria. (a) Distance-based phylogenetic tree of MgtA and MgtB homologs from pathogenic bacteria, computed by FastME implemented in NGPhylogeny.fr from a Clustal Omega multiple sequence alignment and visualized as a circular cladogram. Strains and UniProt accessions analyzed: *Acinetobacter baumannii* MDR-ZJ06 (A0A654L116), *A. baumannii* SDF (B0VRM8), *A. baumannii* ATCC 19606 (D0C699), *Escherichia coli* K-12 (P0ABB8), *E. coli* O44:H18 EAEC (D3GW42), *Yersinia pestis* (A0AAX2HZE9), *Pseudomonas aeruginosa* PAO1 (Q9HUY5), *P. aeruginosa* UCBPP-PA14 (A0A0H2ZH03), *P. aeruginosa* PA7 (A6VCT0), *Salmonella enterica* serovar Typhimurium LT2 (MgtA:

P36640; MgtB: P22036), *S.* Typhimurium 14028s (D0ZTB2), *S. enterica* (A0A0W3KK13), *Klebsiella pneumoniae* (A0A086ICS8 / A0A486UQ81), and *Enterococcus faecium* ATCC BAA-472 (Q3Y0B4). *E. coli* K-12 was included as a non-pathogenic reference. The most divergent pair within this dataset, *E. faecium* ATCC BAA-472 MgtA (Q3Y0B4) and *S.* Typhimurium 14028s MgtA (D0ZTB2), shared 48.1% pairwise sequence identity (calculated in Jalview), indicating substantial conservation despite clear sequence divergence across taxa, consistent with their shared assignment to the Mg^{2+}-transporting P-type ATPase family. **(b)** Distance-based phylogenetic tree of MgtC homologs from pathogenic bacteria, generated by the same procedure as in (a). Strains and UniProt accessions analyzed: *S.* Typhimurium LT2 (P0CI70), *S.* Typhimurium 14028s (D0ZLQ7), *S. enterica* (A0A0M0QP52), *P. aeruginosa* UCBPP-PA14 (paralogs A0A0H2ZHS2 / A0A0H2Z9J0 / A0A0H2ZAK6), *P. aeruginosa* PAO1 (paralogs Q9HVF6 / Q9I0S6 / Q9I1W7), *P. aeruginosa* PA7 (paralogs A6V4Q2 / A6V633 / A6VC28), *K. pneumoniae* IS43 (W1DJ03), *A. baumannii* ATCC 19606 (D0C6A0), and *A. baumannii* ATCC 17978 (A0A077GEF9). Within this dataset, the most divergent pair shared 37.1% pairwise sequence identity (calculated in Jalview). Unlike MgtA/MgtB, MgtC is not classified as a canonical Mg^{2+} transporter but is associated with bacterial adaptation to Mg^{2+} limitation and intracellular survival. The MgtC homologs analyzed here exhibit a narrower taxonomic distribution and greater sequence divergence than MgtA/MgtB, while preserving conserved segments that may underlie shared structural or regulatory features.

Underlying datasets. The full multiple sequence alignments used to construct the trees in panels (a) and (b) are provided as Supplementary Data S1 (MgtA/MgtB alignment) and Supplementary Data S2 (MgtC alignment), respectively. A complementary broad-scale phylogenetic analysis based on MgtA homologs from 2,450 bacterial strains, together with the corresponding sequence metadata, is provided as Supplementary Data S3-1 and Supplementary Data S3-2.
