# Supplementary figures and images for: Magnesium Transporters as Crucial Regulators of Bacterial Survival and Pathogenicity
Source: Microorganisms. 2026 May 1;14(5):1033. doi: 10.3390/microorganisms14051033 (PMC13209247; doi:10.3390/microorganisms14051033)

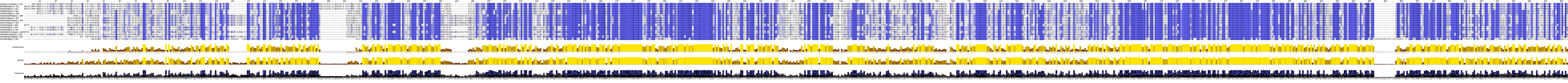

Supplement: Supplementary file 1 [file microorganisms-14-01033-s001.zip › microorganisms-4251839-supplementary/Supplementary_materials/Supplementary_Data_S1.png]

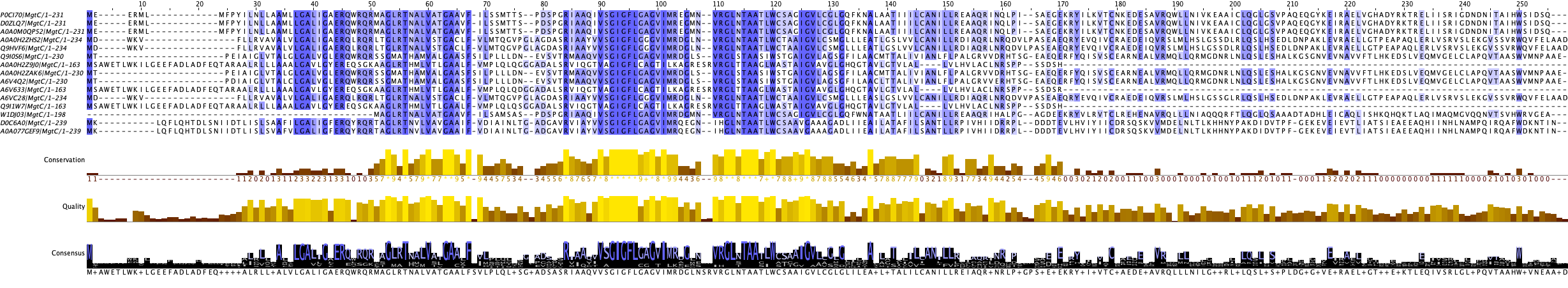

Supplement: Supplementary file 1 [file microorganisms-14-01033-s001.zip › microorganisms-4251839-supplementary/Supplementary_materials/Supplementary_Data_S2.png]

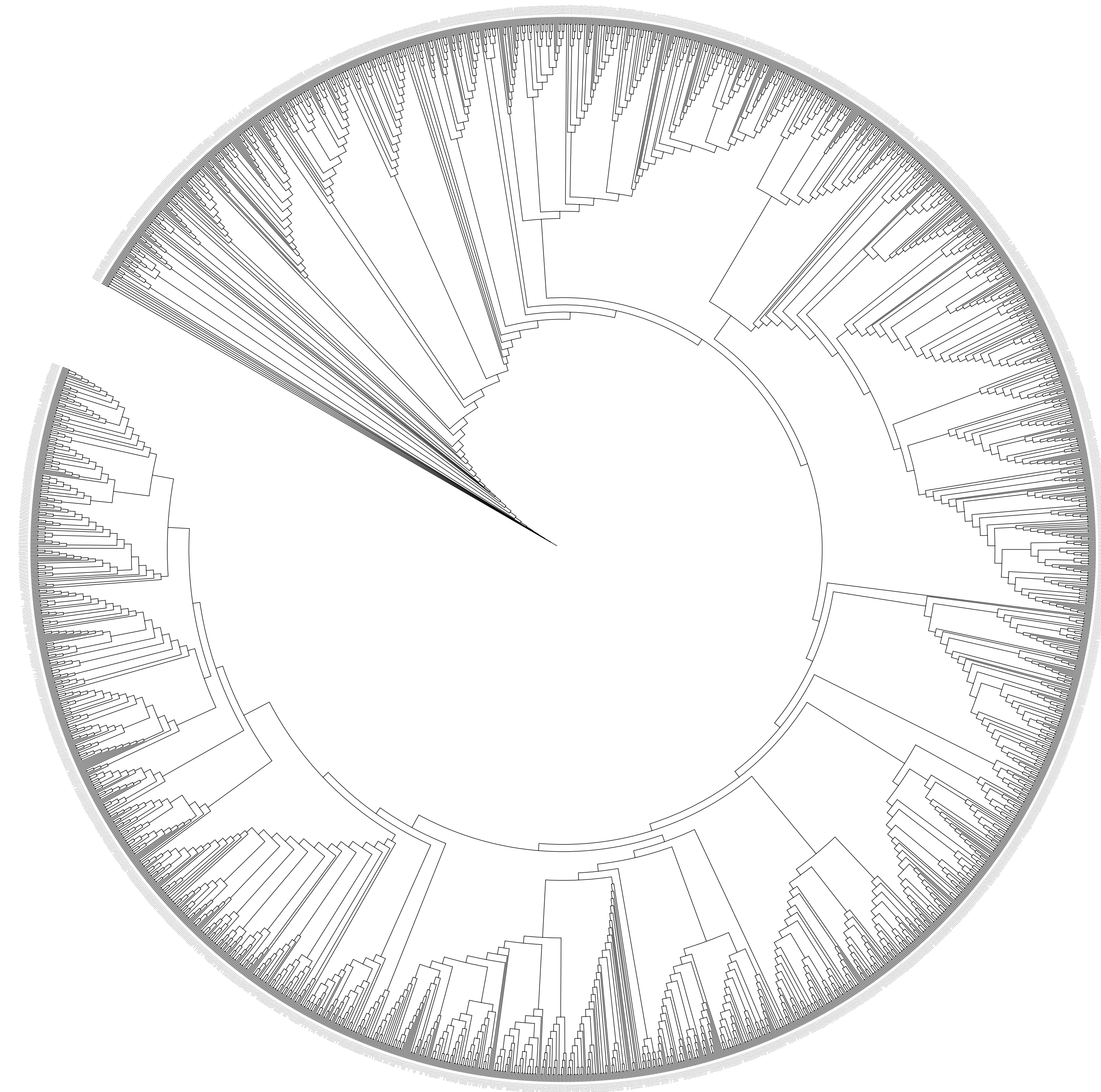

Supplement: Supplementary file 1 [file microorganisms-14-01033-s001.zip › microorganisms-4251839-supplementary/Supplementary_materials/Supplementary_Data_S3-1.pdf]
